# Postmortem Demonstration of the Source of Pulmonary Thromboembolism: The Importance of the Autopsy

**DOI:** 10.1155/2011/108215

**Published:** 2011-10-18

**Authors:** Gina Elhammady, Andrew T. Schubeck, Vicky El-Najjar, Morton J. Robinson

**Affiliations:** Arkadi M Rywlin, M. D. Department of Pathology, Mount Sinai Medical Center and Herbert Wertheim College of Medicine, Florida International University, Miami, FL 3319, USA

## Abstract

Periprostatic or paravaginal venous thromboses are rarely considered clinically as sites of clot origin in patients with pulmonary thromboembolism. The majority of emboli have been demonstrated to originate in the veins of the legs. This report raises awareness of pelvic vein thrombosis as a potential source of pulmonary embolism that is rarely considered or detected clinically, and which usually requires postmortem examination for recognition. It also reviews the possible routes emboli may take to reach the lungs.

## 1. Introduction

Pulmonary thromboembolism is a major cause of death. The majority of emboli have been demonstrated to originate in the veins of the legs. While other sites of clot origin, such as the atrial appendage of the right heart or the vessel wall at the tip of a venous catheter may be suspected based on the clinical presentation, there remains a group of patients for whom the site of clot origin is an enigma clinically. Rarely considered are thromboemboli arising from the pelvic veins, namely, the periprostatic and paravaginal venous plexuses.

We present three male patients in whom periprostatic venous thrombi were the source of fatal pulmonary emboli as demonstrated at autopsy. Additionally, we present one female patient whose source of fatal pulmonary embolism was the paravaginal veins. The pathways for emboli migration from periprostatic or paravaginal veins may be through drainage into the inferior vena cava or via Batson's plexus through paravertebral veins into the superior vena cava. This report highlights a clinically rarely considered source of pulmonary thromboemboli which usually requires postmortem examination for identification and describes the possible pathways of clot migration.

## 2. Case Presentation

### 2.1. Case 1

A 78-year-old male patient experienced syncopal attacks shortly after knee surgery. He was treated for anemia and was sent home. Approximately two weeks later, he was admitted for a second syncopal attack. During his stay, he also experienced a transient seizure and shortness of breath. He developed a persistent hypotension that was refractory to fluids and pressor therapy. The patient eventually developed bradycardia that progressed to pulseless electrical activity. Resuscitative efforts failed. On autopsy, massive bilateral pulmonary embolism was present. “Milking” of the lower limbs failed to produce blood clots from the deep veins of the legs. The periprostatic plexus of veins was found to be thrombosed.

### 2.2. Case 2

**Figure 1:**
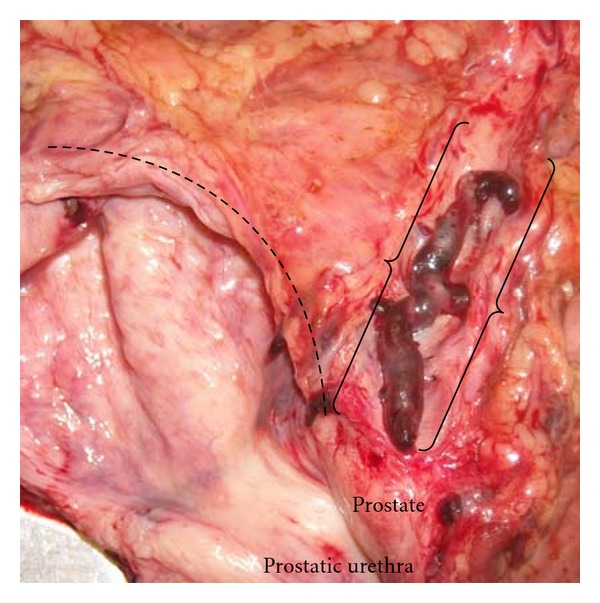
The bladder (dashed line) is open and a dissected periprostatic vein (between brackets) containing a large clot extending into the branches is seen on the right.

A 46-year-old man with a medical history of hypertension, diabetes mellitus, hypercholesterolemia, and an episode of hemolytic anemia, one year prior to presentation, presented with a two-day history of shortness of breath, wheezing, cough, and fever and was found to be hypoxic on arrival. A CT scan of the chest showed extensive bilateral alveolar infiltrates but did not reveal any evidence of pulmonary embolism. The patient developed worsening shortness of breath, hypoxia, and hypotension and required intubation. He suffered a right-sided stroke six days after admission with a CT scan revealing large right posterior cerebral and frontal parietal infarcts. The patient experienced several episodes of cardiopulmonary arrest and expired. On autopsy, massive pulmonary embolism of the midlung and peripheral pulmonary vasculature was present. “Milking” of the legs failed to produce any blood clots. The periprostatic plexus of veins was found to be thrombosed (Figure 1).

### 2.3. Case 3

A 41-year-old HIV-positive man had been admitted and treated for Pneumocystis two weeks prior to presenting to the emergency department with a three-day history of worsening cough and shortness of breath. On admission, he was found to have elevated cardiac enzymes. He later developed two seizure episodes, cardiopulmonary arrest, and expired the same day of admission. Upon postmortem examination, there was massive bilateral pulmonary thromboembolism with pulmonary infarction. Once again, the periprostatic veins were thrombosed.

### 2.4. Case 4

A 57-year-old woman with a previous hysterectomy and gastric bypass surgery (Roux-en-Y) in 2003 presented with abdominal pain and a CT scan that showed a small bowel obstruction. In the OR, the patient was found to have a volvulus involving the Roux limb of the bypass with ischemia of the entire limb and perforation. The ischemic bowel was resected. The patient continued to have abdominal pain and was returned to the OR where multiple small bowel anastomotic leaks were noted with widespread peritonitis. The leaks were repaired, and the necrotic bowel was resected. However, the patient went into septic shock and became unresponsive requiring intubation. A decision to remove supportive life care was made and the patient expired. On autopsy, an embolus was seen in the left main pulmonary artery with extension into the lower lobe with a left lower lobe infarct. “Milking” of the legs failed to produce any blood clots; however, multiple thrombi were identified in the paravaginal veins.

## 3. Discussion

Pulmonary embolism (PE) is a major international health problem and cause of death. Mortality in untreated PE is approximately 30% [1]. Numerous cases are clinically unrecognized, often with fatal outcomes [1]. The prevalence of PE at autopsy (approximately 12–15% in hospitalized patients) has not changed over three decades [1]. 

A contributing factor to the failure to diagnose the disease is that the identification of the source of emboli is often elusive [2]. Clinical and autopsy studies have demonstrated the source of thromboemboli in 50–70% of cases [1]. The majority of emboli have been shown to originate in the veins of the legs, frequently at the level of the femoral and iliac veins [1]. Upper extremity venous thrombosis and thrombi in the superior vena cava, attributed to invasive procedures, may be associated with PE [1, 3, 4]. Cardiac origin of PE as from right atrial or ventricular thrombi plays only a minor role in the overall incidence of the disease [4, 5]. Demonstration of pelvic vein thrombosis as the source of pulmonary thromboemboli is rare. We have been unable to find reports of this site of origin in the recent literature and have found only one abstract of a case which reports the potential of the periprostatic plexus [6]. 

Other sources are difficult to identify both clinically and at autopsy because dissection of the veins behind the knees, calves and soles of the feet is not routinely done after mortem. In addition, thrombus detachment can prevent localizing its point of origin [1]. The postmortem examination for thrombosis of deep veins of the lower limbs typically is confined to the palpation of the soles of the feet and manual compression of the lower extremities with repeated upward compression, “milking,” in an effort to identify premortem clots. This technique may be nonproductive because of inadequate pressure to the leg, particularly in obese patients or because the entire clot may already have migrated. It is therefore important for the autopsy prosector to examine the pelvic venous plexuses for thrombi. 

**Figure 2:**
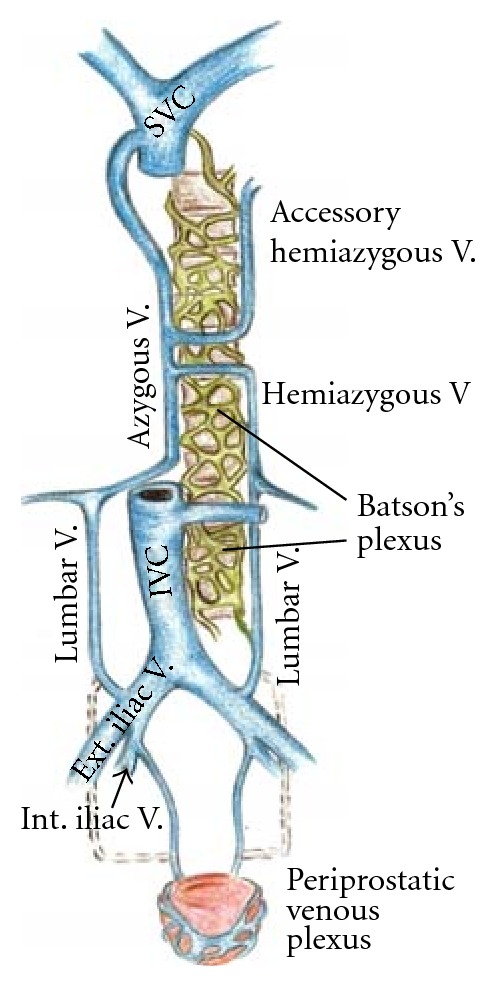
The periprostatic or paravaginal plexus drains primarily to the internal iliac veins via the vesical veins to reach the inferior vena cava and eventually the pulmonary circulation. Alternatively, this plexus communicates with Batson's vertebral plexus which, in turn, reaches the pulmonary circulation via either the azygous system or the superior vena cava. (This figure is the author's illustration).

Rarely is periprostatic or periuterine venous thrombosis considered clinically. Thus, the diagnosis of thrombosis in these sites is most likely to be made only at postmortem examination. It is possible for emboli to arise from these vessels and reach the lungs through two routes as illustrated in Figure 2.

This report raises awareness of a potential source of pulmonary embolism that is rarely considered or detected clinically, and which usually requires postmortem examination for recognition. It also reviews that the possible routes emboli may take to reach the lungs.

##  Disclosure Summary

The authors have nothing to disclose.
